# Supplementary material for: A UMLS-based spell checker for natural language processing in vaccine safety
Source: BMC Med Inform Decis Mak. 2007 Feb 12;7:3. doi: 10.1186/1472-6947-7-3 (PMC1805499; doi:10.1186/1472-6947-7-3)
Supplement: Additional file 13 — RAP application directory. Sets up directory for RAP (RDF application for PHP) source code [file 1472-6947-7-3-S13.gz › rap/doc/phpdoc/utility/IterFind.html]

xml version="1.0" encoding="iso-8859-1"?


Docs For Class IterFind


## Class IterFind

Description

Description |
Methods (details)

Implementation of a find-iterator which delivers statements or quads.

This Iterator should be used in a for-loop like: for($iterator = $memmodel->iterFind(null,null,null,null); $iterator->valid(); $iterator->next()) { $statement=$iterator->current(); };

History: <LI>05-29-2005 : First version of this class.</LI>

- access: public
- author: Daniel Westphal (http://www.d-westphal.de)
- version: V0.9.1

Located in /util/IterFind.php (line 30)

Method Summary

Description |
Methods (details)

IterFind
IterFind
($graph $graph, $subject $subject, $predicate $predicate, $object $object, [$returnAsQuads $returnAsQuads = false])

mixed
current
()

integer
key
()

void
next
()

void
rewind
()

boolean
valid
()

Methods

Description |
Methods (details)

Constructor IterFind (line 106)

Constructor.

$subject, $predicate, and $object are used like find(). $graph has to be a reference to the graph to search in.

- access: public

IterFind

IterFind
($graph $graph, $subject $subject, $predicate $predicate, $object $object, [$returnAsQuads $returnAsQuads = false])

- $graph
  $graph: Resource
- $subject
  $subject: Resource
- $predicate
  $predicate: Resource
- $object
  $object: Resource
- $returnAsQuads
  $returnAsQuads: boolean

current (line 160)

Returns the current item

- access: public

mixed

current
()

key (line 175)

Returns the key of the current item

- access: public

integer

key
()

next (line 148)

Moves Iterator to the next item in the list

- access: public

void

next
()

rewind (line 126)

Resets iterator list to start

- access: public

void

rewind
()

valid (line 138)

Says if there are additional items left in the list

- access: public

boolean

valid
()

Documentation generated on Thu, 7 Jul 2005 13:41:50 +0200 by phpDocumentor 1.3.0RC3
